# Done before needed: The infrastructure that made crystallography so popular for machine learning

**DOI:** 10.1063/4.0000951

**Published:** 2025-10-27

**Authors:** Brian H Toby

**Affiliations:** Argonne National Lab, Lemont, IL, USA

## Abstract

Crystallography, like all other areas of science, attempts to create a body of knowledge. In our case this concerns the structure of the crystalline and more recently, non-crystalline substances, that are found in biological materials, in the earth and space, and those we create for purposes of improving health, commerce or just curiosity. One area where crystallographers have excelled is at curating and making that information available, developing standardized ways of communicating measurements and results, and providing software tools. This, plus the great importance that chemical/biochemical structure offers for understanding the physics of how a substance performs its actions, has made crystallographic information an early target for applications of machine learning technologies. In a parochial fashion, concentrating more on areas where the speaker has been involved, this presentation will cover where having the right tools before it was clear that they would be needed for “AI” purposes enabled major advances in computation. The unanswered question is: how far will these advances bring us?

